# Global public goods and the global health agenda: problems, priorities and potential

**DOI:** 10.1186/1744-8603-3-9

**Published:** 2007-09-22

**Authors:** Richard D Smith, Landis MacKellar

**Affiliations:** 1 Health Policy Unit, Department of Public Health and Policy, London School of Hygiene and Tropical Medicine, London, UK; 2 International Institute for Applied Systems Analysis, Vienna, Austria and Health Economics Centre, City University, London, UK

## Abstract

The 'global public good' (GPG) concept has gained increasing attention, in health as well as development circles. However, it has suffered in finding currency as a general tool for global resource mobilisation, and is at risk of being attached to almost anything promoting development. This overstretches and devalues the validity and usefulness of the concept. This paper first defines GPGs and describes the policy challenge that they pose. Second, it identifies two key areas, health R&D and communicable disease control, in which the GPG concept is clearly relevant and considers the extent to which it has been applied. We point out that that, while there have been many new initiatives, it is not clear that additional resources from non-traditional sources have been forthcoming. Yet achieving this is, in effect, the entire purpose of applying the GPG concept in global health. Moreover, the proliferation of disease-specific programs associated with GPG reasoning has tended to promote vertical interventions at the expense of more general health sector strengthening. Third, we examine two major global health policy initiatives, the Global Fund against AIDS, Tuberculosis and Malaria (GFATM) and the bundling of long-standing international health goals in the form of Millennium Development Goals (MDG), asking how the GPG perspective has contributed to defining objectives and strategies. We conclude that both initiatives are best interpreted in the context of traditional development assistance and, one-world rhetoric aside, have little to do with the challenge posed by GPGs for health. The paper concludes by considering how the GPG concept can be more effectively used to promote global health.

## Background

Although the health of the world's poor has been an apparent humanitarian concern of the world's rich for many years, results based on appeals to such 'humanity' have not been sufficient. Even recent high-profile engagements by the Global Fund Against AIDS, Tuberculosis, and Malaria (GFATM), the United States President's Emergency Plan for AIDS Relief (PEPFAR), the Bill and Melinda Gates Foundation, and WHO disease-targeted programs such as Stop TB and Roll Back Malaria have failed to bring us to the levels of assistance needed to achieve the health-related Millennium Development Goals (MDGs).

Beginning in the late 1990s, the suggestion emerged to address this situation by encouraging policy makers in rich countries to view health assistance not only as humanitarian but as a selfish investment in protecting the health of their *own *populations. The key concept underlying this new interpretation is that of 'global public goods' (GPGs) [1].

This paper briefly outlines and clarifies the GPG concept, and identifies two major health GPGs: health research and development (R&D), and communicable disease control. However, just because a problem is global and formidable, or just because the response is multilateral, does not necessarily mean that it has anything to do with the undersupply of GPGs. We show this by considering two major global health innovations, GFATM and the re-branding of traditional health objectives in the form of MDGs. Based on the review, in a concluding section we suggest three ways in which the GPG concept can be more effectively deployed to promote global health development.

### Problems: Why do 'global public goods' require collective action?

The GPG concept is an extension of the economic tradition of classifying goods and services according to where they stand along two axes: one measuring rivalry in consumption; the other measuring excludability. Pure private goods are those that we are most used to dealing with in our day-to-day lives, and are defined as those goods (like a loaf of bread) that are diminished by use, and thus rival in consumption, and where individuals may be excluded from consuming them. At the opposite end of the spectrum are pure public goods, which are non-rival (not diminished by use) and non-excludable (if the good is produced, it is freely available to all). Public security is an often-cited example. In between these extremes are 'impure' goods, such as 'club goods', which have low rivalry but high excludability, and 'common pool goods', which have low excludability but high rivalry [2]. Clearly in this case, 'health' itself is a private good, as are the majority of goods and services used to produce health [3].

One of the fundamentals of public economics is that the free market – the interplay of individual supply and demand decisions mediated through the price system – will result in the provision of less than the collectively optimal level of *public *goods. Thus, the state has a role to play, either in producing the good directly (the traditional approach) or at least in arranging for its production by a private firm (the increasingly popular 'outsourcing' strategy). Examples of national public goods run from police protection to national security to financial regulation to museums and artistic ensembles. But some goods are quite clearly public at the global level. The classic case is greenhouse gas emission control.

A reasonable functional definition would be that a GPG is "a good which it is rational, from the perspective of a group of nations collectively, to produce for universal consumption, and for which it is irrational to exclude an individual nation from consuming, irrespective of whether that nation contributes to its financing" [[3], page 9]. The main issue facing non-national (global or regional) public good provision is how to ensure collective action in the absence of a 'government' to directly finance and/or provide the public good, the response in the case of national public goods [4].

Given the reluctance of voters to support programs some of whose benefits are felt beyond the borders, an aspect deserving special attention is mobilizing non-traditional sources of finance [5]. Cutting across all aspects of the GPG concept is the key fact that collective action is in donor countries' self-interest.

The GPG concept thus has a specific meaning within economics. However, it has suffered as it has found currency as an advocacy tool for global resource mobilisation [6-8]. Since a GPG calls for collective action, then, clearly, one's favourite program must be producing a GPG. This has given rise to "fuzziness" and "trendiness" [[9], page 2). The GPG 'tag' is at risk of being attached to anything of particular attraction and importance, to the point that, at the limit, anything promoting development could be considered a GPG. This is to be avoided, as overstretching the concept devalues the validity of the point that there really is a class of GPGs requiring public support or provision [10].

## Priorities I: What GPG areas represent priorities in global health?

Indeed, Smith et al [11] suggest that the GPG concept may perhaps be most usefully applied to just two aspects of health. The first is research and development (R&D) and the second is communicable disease control (epidemiological surveillance, immunization, and other preventive measures). In the next section, we ask how the GPG concept, particularly the need for collective action, has affected policies and programs in these areas.

### Health research and development

Health R&D unquestionably has GPG aspects, and there is not enough of it in fields that would benefit poor countries. Historically, the public and the not-for profit sectors have carried out research resulting in new drugs and treatments, but the private for-profit sector now plays the largest role [12,13]. An important policy question is therefore how to encourage private sector firms to engage in research benefiting poor countries and peoples: the ubiquitous '90-10 problem' (that 90% of global R&D spending in health is targeted at diseases affecting only 10% of the world's population) [14]. A related but distinct question is how to bolster the demand for drugs in low-income countries (and hence firms' willingness to engage in R&D); we touch on this in a section below in which we discuss Advance Purchase Commitments and other financial innovations.

A GPG perspective would argue that provision of adequate R&D related to diseases of the poor requires innovative collective action. This no-more-business-as-usual attitude has clearly motivated the explosion in the number of Global Public-Private-Partnerships (GPPPs) undertaking R&D related to diseases of the poor [15-17]. An order-of-magnitude estimate of GPPPs' annual spending might be US$1 billion [18]. This may be compared with total global health R&D spending on the order of US$100 billion [14]. This sounds small, but when the US$1 billion is compared to the estimated US$2.5 billion spent on health R&D by governments in low-and middle-income countries, the perspective is much more favourable for the important role played by GPPPs. In the area of R&D related to "neglected diseases" of the tropics, GPPPs occupy a decisive position.

### Communicable disease control

The GPG perspective supports collective action in the area of infectious disease control when reduction in disease prevalence in Country A has a benefit for Country B as well. Areas in which this is particularly true are diseases for which eradication is feasible (polio) and diseases that are highly transmissible around the world, whether by human carriers (SARS), by trade in products (BSE), or by animal vectors (West Nile Virus, avian influenza). The control of antibiotic resistance is a closely related GPG problem.

As in the case of R&D, the GPG perspective has informed a number of major new initiatives to provide (not only develop new means of), communicable disease control. These include the GAVI Alliance (formerly the Global Alliance for Vaccines and Immunization), Stop TB, Roll Back Malaria, and others. The main question such initiatives face is the form that assistance should take.

There are basically three types of public health interventions: vertically targeted interventions (focused immunization or disease eradication campaigns, for example), horizontally targeted interventions (universal access to a basic medical care package including vaccination, for example), and sector-wide interventions such as capacity building for improved infrastructure and administration. For many years, donors and health officials in low- and middle-income countries gave emphasis to vertical interventions financed by 'earmarked' funds. Problems with this approach include duplication and lack of coordination among projects, 'recipient fatigue' in health ministries forced to administer multiple grants, distortions in local resource allocation such as poaching skilled personnel, and "crowding out" (of which more below) [19]. All donors and the partner countries have committed themselves, through the 2005 Paris Declaration, to pursue harmonisation of practices, standards, and criteria in foreign assistance. Yet the urge to compete rather than cooperate is strong, and well-entrenched donor practices and protocols disappear only stubbornly.

One of the ironies of the current health landscape is that, as many in the public health community moved away from vertical interventions towards broader approaches the GPG perspective has helped to fuel the proliferation of specific infectious disease-targeted programs. Yet the experiences of programs in immunization, malaria control, and tuberculosis control demonstrate that impacts are limited by sector-wide weaknesses such as lack of a cold chain, shortages of skilled personnel, insufficient resources for operating vehicles, etc. The plethora of new vertical initiatives may contain the seeds of its own failure if health systems are not generally strengthened (including the crucial human resources aspect) [20]. The danger is that the GPG agenda will promote focused interventions easy to "sell" to voters at home because they address an identifiable menace, at the expense of broader health system strengthening. One response is to identify the health system as a prime 'access good' – not a GPG itself, but a fundamental requirement for the provision of GPGs [11].

### Additionality and innovative financing

The GPG perspective has contributed to a large number of new programs. However, some caveats are in order. Providing an adequate supply of a GPG requires spending more than would have been spent in the absence of collective action, i.e. "additionality." The additionality debate is complex, and involves at least three questions:

- At the individual country level, we may ask whether international assistance for production of GPGs in Country X reduced (or, in development parlance "crowded out") Country X's government spending for production of GPGs. Since most of the countries in question are very poor and local needs take clear precedence over global ones, it is probably safe to answer this question in the negative.

- More pressing is the question whether international assistance for production of GPGs in Country X reduced international assistance for production of non-GPGs in Country X. A direct answer to this question is difficult to provide because data on foreign assistance at the level of destination countries are much worse than data by country of origin. Reisen et al [21], looking at the latter, concluded that the average bilateral donor's allocation of US$1 to GPG production reduced its spending on non-GPG foreign assistance by US$0.25.

- Finally, additionality questions may be posed as between donors. The focus of this particular storm concerns the activities of the GFATM and PEPFAR. Many have complained that PEPFAR diverts U.S. resources away from GFATM, a multi-lateral agency, to a bilateral and highly politicised program. Questions of donor additionality are not confined to HIV/AIDS. Cohen [17], in looking at the involvement of GPPPs in health R&D, reports that, in expert interviews, researchers complain that the new availability of philanthropic funds for medical research is "crowding out" funding that would have been received from government agencies such as the U.S. National Institutes for Health.

While the additionality of resources is ambiguous, we can answer a closely related issue definitively. Resources being used for the provision of GPGs in the area of health R&D and communicable disease control come from traditional, not innovative, sources. The typical new initiative depends on a philanthropic institution for its start-up, followed by infusions of government support channelled through bilateral aid organizations [15]. Yet, the GPG concept is firmly rooted in the self-interested use of domestic monies and as such sees funding as distinct from current aid and philanthropic flows. While there have been innovative fund raising suggestions ranging from a tax on airline travel to a global lottery, progress has been slow. In the health field, Advance Purchase Commitments and the "front-loading" of international assistance for immunization via the GAVI Alliance's International Finance Facility) represent steps forward [22]. In the first case, donors pre-commit to vaccine purchases if R&D is successful; in the second, future aid commitments are collateralized so that funds can be raised immediately in the international bond markets. These are welcome innovations, but they still tap the same source: international donor agency budgets.

## Priorities II: What priority areas in global health do not represent GPGs?

Inverting the logic above, in this section we wish to clarify that a number of the major priorities in global health today do *not *represent GPGs. This does not diminish their importance, but it means that progress in these areas should not be equated with progress regarding the undersupply of GPGs. The two innovations we review are the GFATM and the re-framing of traditional health goals in the form of time-bound Millennium Development Goals.

### The Global Fund to Fight AIDS, Tuberculosis and Malaria (GFATM)

Of the three scourges fought by the GFATM, only tuberculosis can be said with accuracy to represent a global public "bad." Malaria has significant cross-border aspects, but these make malaria control a regional public good (and one requiring collective action at the regional level), not a global one. Apart from R&D aspects, the public good problems associated with HIV/AIDS are regional at most, not global [23]. Despite inflammatory rhetoric heard at the beginning of the pandemic, HIV/AIDS has not proven to be a disease that spreads globally like SARS or pandemic influenza. Obvious cross-border aspects like transmission associated with long-haul truckers and migrant workers in Southern Africa call for a regional, not a global response.

Even if its transmission *did *qualify HIV/AIDS as a GPG, the GFATM response is not dealing with the disease using GPG logic. The main concern of GFATM (and PEPFAR, and the WHO's "Three by Five" program, and the G8 nations' commitment to universal access by 2010) is the provision of subsidized antiretroviral therapy (ART) to AIDS sufferers in low-income countries. ART is rival (therapy made available to one person or nation cannot be made available to another) and excludable (persons can be barred from receiving it). By contrast, AIDS prevention, in the form of media campaigns, condom distribution, voluntary counselling and testing, reduction of sexually transmitted infections, and encouragement of male circumcision, is non-rival (if A remains HIV-negative as a result of a prevention program, his sex partners B and C are protected equally) and non-excludable (no one can prevent C from enjoying the same protection as B).

Another argument runs that the destabilizing effect of HIV/AIDS in seriously affected countries gives rise to global impacts, but so do the destabilizing effects of everything else, like unemployment and hunger, that contribute to misery. And again, even it the disease's destabilizing potential *did *qualify it as a GPG, the international policy response does not prioritize GPG aspects. In a world of finite resources, the provision of ART in low-income countries must come at the expense of prevention (if resources were not finite, of course, such tragic choices would not need to be made). The lopsided cost-ineffectiveness of ART means that each disability-adjusted life year (DALY) saved by treatment comes at the expense of many, many more DALYs lost in the future because the prevention measures needed to reduce transmission cannot be implemented [[24], p. 139]. If it were the looming scale of the AIDS catastrophe and its global spill over effects that were of greatest concern to donors, they would give priority to prevention, not treatment.

The need for collective action against AIDS was not the driving force behind the founding GFATM. It was born rather of frustration, especially on the part of AIDS activists, that good ideas from the field were not receiving deserved support because of donor red tape [25]. The response was to be a funding agency which would not assess proposals itself, relying rather on an independent panel, and would use local accounting firms to monitor implementation. Its comparative advantage would be focusing resources quickly on 'best shot' programs in countries with greatest need, as well as raising the profile of the disease. The hands-off approach to program formulation and implementation, it was argued, would mean that the GFATM would have no agenda of its own; aid-recipient countries would be able (through their representation on the review panel) to set their own priorities. The absence of a programmatic/operational agenda would allow the Fund to concentrate on mobilising and disbursing resources [26]. In sum, the rationale for the GFATM was not so much that a GPG was being undersupplied, but that assistance was not being provided efficiently or in a way consistent with needs.

Even if the GFATM is interpreted as a bold initiative – and not all do so, since most proposals still come through governments – we run into the caveats made above. The Fund may have generated additional resources or it may not have – the fact that it has struggled against resource constraints practically since its inception [26] gives some reason to suspect the latter. So do anecdotes making the rounds that the arrival of GFATM in some countries has led bilateral donors (apart from the US, through PEPFAR) to limit their own AIDS efforts. GFATM has not mobilized non-traditional sources of finance. As of mid-2007, out of the US$10.9 billion cumulatively pledged for all three diseases through 2008, only US$707 million comes from private firms, foundations, and individuals; this consists almost in its entirety of a US$650 million grant from the Bill and Melinda Gates Foundation. With its resources being pledged almost entirely from traditional donor countries' aid budgets, the GFATM is replicating the source-structure of existing aid flows.

A word on the U.S. PEPFAR is in order. PEPFAR promises US$15 billion (US$9 billion of which are claimed to represent new resources) for the global fight against AIDS but only allocates US$1 billion to GFATM [27]. Over half of the resources are targeted to providing AIDS treatment. Of funds allocated to prevention, a significant proportion is earmarked for faith-based programs encouraging teenaged sexual abstinence and discouraging multiple-partner sex. The ability of PEPFAR to finance activities benefiting key target populations – commercial sex workers and injecting drug users – is tightly constrained by law. It is hard to consider PEPFAR as collective provision of a GPG when the resources it makes available could have been channelled through a genuinely collective institution (GFATM) and when its prevention programs are designed to cater to a domestic political constituency.

### The Millennium Development Goals for Health

The main focus of global health development at present is the health Millennium Development Goals (MDGs) (General Assembly, A/55/L.2, September 18, 2000).

The targets associated with the health MDGs are to: (i) reduce child mortality by two-thirds between 1990 and 2015; (ii) reduce the maternal mortality ratio by three quarters between 1990 and 2015; (iii) halt and begin to reverse the spread of AIDS by 2015; and (iv) halt and begin to reverse the incidence of malaria and other major diseases by 2015. Developed countries and the development agencies will, in return for low- and middle-income countries' devoting effort to attaining the MDGs, take primary responsibility to establish a global partnership for development. In the area of health, the associated target is: (v) provide, in cooperation with pharmaceutical companies, access to affordable essential drugs in developing countries.

How do MDGs for health relate to the GPG perspective? Some of them, for example, those related to tuberculosis and access to drugs (or at least the R&D aspect of that problem), address GPG problems directly. Others, for example those related to maternal mortality, most child mortality, and HIV/AIDS, respond to humanitarian concerns, not GPG problems.

As in the case of the GFATM, when we look carefully at the origins of the MDG approach, we find that GPG logic is absent. The MDGs emerged from profound dissatisfaction with the effectiveness of aid to date and insistence on a "results focus" and improved monitoring and evaluation [28]. This process is the elaboration of a Poverty Reduction Strategy Paper or PRSP [29]; the PRSP process, in turn is meant to encourage countries to adopt a long-term vision "by bringing out explicit awareness of poverty issues and promoting participation of stakeholders" [[29], page 11). PRSPs are meant to be country-driven ('ownership'), results-oriented, and participatory; reflect input of civil society and the private sector [30]. Countries are meant to prioritize the MDGs in accordance with their long-term vision of development needs. The PRSP process is also meant to force explicit linkages between fiscal resource allocation decisions and poverty reduction through the putting-in-place of Medium-term Expenditure Frameworks or MTEFs [31].

"We will recognize country ownership and a partnership of equals," runs the donor governments' position, "if you will deliver results and ensure stakeholder participation." "We will deliver results and ensure stakeholder participation," runs beneficiary governments' position, "if you will acknowledge country ownership and a partnership of equals." This is a laudable win-win outcome – but it responds to a crisis in traditional development assistance, not to the need for collective action to supply GPGs. "Country ownership" and acceding to equal partnership are the last things that would be stressed in an approach built on GPG logic. Far from encouraging donor-country voters to support generous foreign aid programs because they are in their own interest, these discourage them from doing so [25].

To conclude, the two initiatives examined show that massive mobilization of humanitarian assistance in pursuit of common goals should not be confused with collective action to ensure the adequate supply of GPGs. That observation does not lessen the importance of such actions, but it guards us against the fallacy of concluding that, just because there is a multiplication of high-profile innovations, fundamental GPG problems are being effectively addressed.

## Potential: How can the GPG perspective best be used to promote global health?

The GPG concept, discovered by the aid community in the late 1990s, can be a powerful tool in promoting global health because it marshals arguments of self-interest. It can be used to identify areas in which global collective action is needed, specify where the costs and benefits will rest and communicate to the public why spending to promote health thousands of kilometers around the world is not a waste of their tax dollars. Yet, we find that the GPG perspective has been a mixed blessing.

We looked at two acknowledged GPGs related to health, namely R&D and communicable disease control. While recognition of the need for global collective action has supported a large number of new initiatives, it remains to be determined what the result is in terms of additional funds. Those funds that have been generated have come from traditional philanthropic and public sources. The proliferation of infectious disease initiatives has promoted a vertical, "stovepipe" approach, to the detriment of broad health sector strengthening.

We then looked at two of the major global innovations in health, the GFATM (and, closely related, PEPFAR) and the re-packaging of traditional health concerns in the form of MDGs. We concluded that both can be more easily understood as addressing weaknesses in traditional humanitarian aid – red tape, lack of country ownerships, insufficient stakeholder involvement, need for results-based management, etc. – than as addressing problems of GPG provision.

All of the new initiatives we have discussed here, and many that we have not mentioned, are funding or doing valuable work. How might the GPG perspective strengthen them and lead to other efforts, as well?

First and foremost, within existing programs and when proposing new ones, the aid community should adhere to the strict economic definition and avoid the temptation to use the GPG 'tag' as a general-purpose fund-raiser. If we focus GPG logic on those goods and services where global collective action really is needed, that action is more likely to be achieved. Where humanitarian grounds, not rational self interest, are the main motivation for action – as in providing subsidized treatment for AIDS sufferers in poor countries – we should say so without equivocation. Where general health system strengthening is required to guarantee access to GPGs such as immunization or tuberculosis control, this should be stated explicitly, even if it means that budgets for GPG provision strictly defined may be reduced as a result.

Second, the aid community should stress to policy makers that, where the GPG label is appropriate, as in the case of communicable disease control, what is needed is not only new packaging/labeling of existing resources, but resources *additional *to those already being made available, which means mobilizing *innovative sources of financing*. The current elevated level of concern over emergent diseases, including pandemic influenza, is an ideal context in which to press for a more pro-active response. So is the rapid development of financial engineering tools related to aid, such as advance purchase commitments, collateralization future aid commitments in the bond market so as to "frontload" aid, etc.

Third, the relative ease of financing disease-specific actions, as opposed to broad sector strengthening, should not be allowed to distort health sector policy or dictate the structure of support. Where sector support serves an "access" function, the argument that it is a prerequisite for provision of GPGs (essentially, communicable disease control) can be used to strengthen its claim on resources.

The aim of this paper was to provide an introduction to the key concepts, and to consider some innovative developments in global health from the GPG perspective. Hopefully this has illustrated the potential and limitations of the concept, and provided a foundation for further discussion of these.

## Competing interests

The author(s) declare that they have no competing interests.
